# Self-reported prevalence and awareness of metabolic syndrome: findings from SHIELD

**DOI:** 10.1111/j.1742-1241.2008.01770.x

**Published:** 2008-08

**Authors:** S J Lewis, H W Rodbard, K M Fox, S Grandy, for the SHIELD Study Group

**Affiliations:** 1 Northwest Cardiovascular InstitutePortland, OR, USA; 2 Endocrine and Metabolic ConsultantsRockville, MD, USA; 3 Strategic Healthcare SolutionsLLC, Monkton, MD, USA; 4 AstraZeneca LPWilmington, DE, USA

## Abstract

**Purpose:**

This study assessed awareness of metabolic syndrome and evaluated health knowledge, attitudes and behaviours of respondents at risk.

**Methods:**

Study to Help Improve Early evaluation and management of risk factors Leading to Diabetes (SHIELD), a longitudinal US population-based survey initiated in 2004, included respondents, ≥ 18 years of age, reporting a diagnosis of metabolic syndrome. Prevalence of metabolic syndrome was compared in SHIELD and National Health and Nutrition Examination Survey (NHANES) 1999–2002 survey. The proportion of SHIELD respondents who had heard of and/or understood metabolic syndrome was estimated. Respondents at high risk for metabolic syndrome were stratified into attitude-behaviour categories of ‘Already Doing It’, ‘I Know I Should’ and ‘Don't Bother Me’ and differences in attitudes and behaviours were evaluated with chi-square tests.

**Results:**

Prevalence of reported metabolic syndrome was 0.6% in SHIELD screening questionnaire respondents (*n =*211,097) vs. 25.9% in NHANES (*n =*10,780). Less than 15% of SHIELD baseline questionnaire respondents (*n =*22,001) had heard of or understood metabolic syndrome. Attitudes toward health status were more favourable in the ‘Doing’ group (27% reported fair/poor health) compared with those in the ‘Should’ (38%) and ‘Don’t’ (54%) groups (p < 0.0001). The ‘Don’t’ group was most likely to prefer medications to lifestyle change (13% vs. 2–4%) compared with ‘Should’ and ‘Doing’ groups (p < 0.0001). More ‘Doing’ respondents (79%) than ‘Should’ (59%) and ‘Don’t’ (48%) respondents reported exercising regularly (p < 0.0001).

**Conclusions:**

The lack of knowledge about metabolic syndrome reported in SHIELD indicates limited penetration of this concept into public awareness. With behaviour categories, respondents who report healthy attitudes are more likely to embrace lifestyle changes, while respondents who do not care may be more difficult to treat.

**What's known**

Metabolic syndrome, a constellation of cardiometabolic risk factors, is associated with increased risk for cardiovascular disease (CVD) and diabetes mellitus. Interventions to prevent or manage dyslipidaemia, hypertension, insulin resistance and abdominal obesity have been shown to reduce the CVD risk.

**What's new**

Patient knowledge, attitudes and behaviour play a large role in preventing and managing the risk factors comprising metabolic syndrome. The present study provides insight into the health-related attitudes and behaviours of respondents with or at risk for metabolic syndrome, including awareness and understanding of the syndrome. Improved understanding of self-reported attitudes and behaviours may facilitate more effective communication with physicians and intervention strategies for patients at risk for diabetes and cardiovascular disease.

## Introduction

The presence of metabolic syndrome is associated with increased long-term risk for both atherosclerotic cardiovascular disease (CVD) and type 2 diabetes mellitus, and should thus be addressed in clinical practice (1,2). The National Cholesterol Education Program (NCEP) Adult Treatment Panel III (ATP III) report defined metabolic syndrome as the following constellation of risk factors: dyslipidaemia, hypertension and insulin resistance, abdominal obesity, as well as an inflammatory and prothrombotic state (2), with each metabolic syndrome component associated with heightened cardiovascular risk (1,3). ATP III, the National Heart, Lung and Blood Institute, and the American Heart Association have identified specific underlying risk factors for metabolic syndrome, including obesity, physical inactivity, atherogenic diet, cigarette smoking, and family history of premature coronary heart disease (2,3). However, other well-defined factors beyond the clinical criteria that define metabolic syndrome, such as patient knowledge, attitudes and behaviour, may contribute to the development of metabolic syndrome as well as to the diseases to which it predisposes.

Because its components are readily measurable in clinical practice, metabolic syndrome provides an opportunity for clinicians to assess risk during a standard office visit. However, identifying metabolic syndrome is just the first step. Preventing CVD requires that patients possess adequate knowledge and awareness of the syndrome's diagnostic criteria, its implications for long-term disease risk, and the behaviours required to reduce such risk. The conundrum, however, is that while patients may be aware that they have the conditions that compose metabolic syndrome, they may not understand that these conditions put them at high risk for CVD and diabetes. Furthermore, the transition from understanding risk to moderating it is hampered by patient-specific barriers that must also be identified and managed. Patient education alone often does not result in health-enhancing behaviour change, as risk reduction typically requires sustained lifestyle modifications.

The Study to Help Improve Early evaluation and management of risk factors Leading to Diabetes (SHIELD) is a large, longitudinal US population-based survey that provides a unique opportunity to gain further insight into the health-related attitudes and behaviours of respondents with or at risk for metabolic syndrome. The objectives of this investigation were to determine whether individuals were aware of and understood metabolic syndrome and to evaluate knowledge, attitudes and behaviours among those at high risk of metabolic syndrome. It is hoped that improved understanding of these attitudes and behaviours will facilitate more effective clinically based communication and intervention strategies for patients at risk for diabetes and CVD.

## Methods

### SHIELD questionnaire

A screening questionnaire was mailed in April 2004 to a stratified random sample of 200,000 US households, representative of the US population for age of head of household, income, household size, urban density and census region, identified by the Taylor Nelson Sofres (TNS) National Family Opinion panel (Greenwich, CT). The screening questionnaire consisted of 12 questions designed to identify individuals with diabetes and those with risk factors for diabetes. A response rate of 64% (*n =*211,097 adults ≥ 18 years of age) was achieved. A detailed baseline questionnaire was then mailed in September 2004 to 22,001 screened individuals who were identified with diabetes or risk factors related to diabetes. The baseline questionnaire assessed comorbid conditions, health status, knowledge, attitudes, current behaviours related to general health and diabetes, exercise, diet and weight loss. A response rate of 80% was achieved. A detailed description of the SHIELD methodology has been published elsewhere (4,5).

Respondents were classified according to diagnosis of diabetes (type 1 or type 2) and risk factors associated with increased risk of type 2 diabetes. Recognised cardiometabolic risk factors, derived from the literature, national guidelines and expert opinion (2,6), included: (i) abdominal obesity (defined as waist circumference ≥ 97 cm for men and ≥ 89 cm for women), (ii) body mass index (BMI) ≥ 28 kg/m^2^, (iii) diagnosis of dyslipidaemia (cholesterol problems), (iv) diagnosis of hypertension (high blood pressure) and (v) diagnosis of CVD (defined as one or more of heart disease, myocardial infarction, narrow or blocked arteries, stroke, coronary artery bypass graft surgery, angioplasty, stents or surgery to clear arteries). Stepwise logistic regression analyses verified that these five cardiometabolic risk factors were independently and equally predictive of diabetes diagnosis. Respondents with zero, one or two of the five risk factors were further classified as low risk for diabetes, and respondents with 3–5 risk factors were classified as high risk.

For metabolic syndrome, SHIELD respondents were asked if they had ever been told by a doctor, nurse or other health professional that they had metabolic syndrome or syndrome X. The proportion of respondents reporting a diagnosis of metabolic syndrome was estimated as the prevalence of metabolic syndrome in SHIELD. Previous panel surveys have been used to calculate the population prevalence of conditions such as migraine (7) and bipolar disorder (8).

### Assessing knowledge, attitudes and behaviours

Knowledge, attitudes and behaviours of respondents in the high-risk group were evaluated in the context of a system used by the American Dietetic Association (ADA) (9). SHIELD respondents with 3–5 cardiometabolic risk factors (high risk) were assigned to one of the following three groups derived from ADA-defined behaviour categories:

- ‘Already Doing It’ (Doing), consisting of people who are concerned about diet, nutrition and fitness, and have taken significant actions to change their eating patterns and lifestyles in accordance with these concerns.
- ‘I Know I Should, but…’ (Should), consisting of people who are concerned about the above issues, but have not taken significant actions to address their concerns.
- ‘Don't Bother Me’ (Don’t), consisting of people who are not concerned about their diet, overall nutrition and fitness.

Respondents were assigned to one of these groups based on their response to an attitude question in the SHIELD baseline questionnaire; respondents rated their level of agreement with the statement ‘I don't even bother to try and stay healthy’. Those who agreed strongly or somewhat were assigned to the Don't group, while those who disagreed strongly or somewhat were assigned to the Doing group. Lastly, those who responded with ‘Neither agree nor disagree’ were assigned to the Should group. Differences in health knowledge, attitudes, and behaviours, including diet, exercise and medication-taking behaviour, were assessed across the three groups.

### Statistical analyses

Data from the SHIELD questionnaire were compared with data derived from the 1999–2002 National Health and Nutrition Examination Survey (NHANES, *n =*10,780) for estimating the prevalence of metabolic syndrome. NHANES includes self-reported diagnosed conditions as well as clinical evaluation and laboratory testing to confirm diagnoses and to identify undiagnosed conditions (10,11). Individuals, ≥ 18 years old, were considered to have metabolic syndrome in NHANES if clinical criteria and laboratory test results indicated at least three of the following factors: waist circumference ≥ 102 cm (40.2 inches) in men or ≥ 88 cm (34.6 inches) in women, serum triglycerides ≥ 150 mg/dl, high-density lipoprotein cholesterol < 40 mg/dl in men or <50mg/dl in women, blood pressure ≥130/85mmHg or fasting serum glucose ≥ 110 mg/dl according to the NCEP ATP III diagnostic criteria (2). Prevalence estimates from SHIELD and NHANES were computed stratified by age group and gender. Differences among age groups and gender were tested using chi-square test.

Comparisons across groups (type 1 and type 2 diabetes, high risk, low risk) for knowledge of metabolic syndrome were made using ANOVA test. Comparisons across the ADA behaviour categories (‘Doing’, ‘Should’ and ‘Don’t’) for knowledge, attitudes and behaviours were computed using ANOVA tests. Statistical significance was set *a priori* as p < 0.01 as multiple comparisons were made.

## Results

### Metabolic syndrome prevalence

In the SHIELD screening questionnaire, only 0.6% of the total population (*n =*211,097) reported a metabolic syndrome diagnosis. In contrast, NHANES (*n =*10,780) data using clinical and laboratory criteria indicated a metabolic syndrome prevalence of 25.9% in the adult population (Table 1). The SHIELD respondents and NHANES participants were similar to the US Census population, with 52% of all three samples being aged 18–44 years; 51.8% SHIELD, 50.8% NHANES, 51.9% US Census were women; and 86.7% SHIELD, 71.7% NHANES, and 82.5% US Census were white. For household income, 39.1% SHIELD, 38.0% NHANES and 38.9% US Census had incomes < $40,000 annually.

**Table 1:** Prevalence estimates for metabolic syndrome from SHIELD and NHANES, by age and gender

	Men		Women		Total	
	SHIELD (*n* = 99,216)	NHANES (*n* = 5395)	SHIELD (*n* = 111,881)	NHANES (*n* = 5385)	SHIELD (*n* = 211,097)	NHANES (*n* = 10,780)
Age 18–44	0.2	16.0	0.7	16.4	0.5	16.2
Age 45–64	0.4	33.7	1.1	33.7	0.8	33.7
Age 65+	0.4	37.0	0.8	47.3	0.6	42.9**
All ages	0.3	24.4	0.9*	27.5*	0.6	25.9

* p < 0.001 for men vs. women for all ages.

** p < 0.0001 for comparison across three age groups for total samples. SHIELD, Study to Help Improve Early evaluation and management of risk factors Leading to Diabetes; NHANES, National Health and Nutrition Examination Survey.

National Health and Nutrition Examination Survey results indicated that metabolic syndrome was more prevalent among older respondents; 43% for ≥ 65 years of age vs. 16% for ages 18–44 (p < 0.0001). However, self-reporting of metabolic syndrome did not vary widely across age groups in SHIELD. Women were more likely than men to report a metabolic syndrome diagnosis in SHIELD and NHANES (p < 0.001) (Table 1).

### Metabolic syndrome awareness

For SHIELD, there were 368 respondents with type 1 diabetes, 3849 with type 2 diabetes, 5389 at high risk and 5673 at low risk. Type 2 diabetes and high-risk respondents were more likely to be older, white or male and to have lower incomes than respondents with type 1 diabetes or low risk (p < 0.0001) (Table 2).

**Table 2:** SHIELD respondent characteristics for baseline questionnaire

Characteristics	Type 2 diabetes (*n* = 3849)	Type 1 diabetes (*n* = 368)	High-risk (3–5 risk factors) (*n* = 5389)	Low-risk (0–2 risk factors) (*n* = 5673)
Age, **years**, *n* (%)*				
18–44	489 (13)	232 (63)	951 (18)	2732 (48)
45–64	1905 (50)	128 (35)	2448 (45)	2023 (36)
≥ 65	1455 (38)	8 (2)	1990 (37)	918 (16)
Females, *n* (%)*	2219 (58)	227 (62)	3052 (57)	3713 (65)
Whites, *n* (%)	3268 (85)	333 (90)	4758 (88)	5008 (88)
Household income < $40,000*	2020 (52)	155 (42)	2510 (47)	2067 (36)
Geographic region, *n* (%)*				
Northeast	758 (20)	64 (17)	1060 (20)	1068 (19)
South Atlantic	813 (21)	62 (17)	1080 (20)	1005 (18)
Central	1576 (41)	146 (40)	2264 (42)	2383 (42)
Mountain	217 (6)	40 (11)	313 (6)	407 (7)
Pacific	485 (13)	56 (15)	672 (12)	810 (14)

* p < 0.001 for ANOVA across all four groups.

In SHIELD, no significant differences were observed between groups (type 1 and type 2 diabetes, high risk and low risk) regarding awareness or knowledge of metabolic syndrome (p > 0.05), but awareness was consistently low across all groups (Table 3). Across type 1 diabetes, type 2 diabetes, high-risk and low-risk respondents, 13–14% indicated they ‘have heard about’ metabolic syndrome, and 9–11% reported that they ‘understand what’ the syndrome is.

**Table 3:** Awareness of metabolic syndrome among SHIELD respondents from the baseline questionnaire

Proportion of respondents agreeing strongly or somewhat	Low risk: 0–2 risk factors (*n* = 5673)	High risk: 3–5 risk factors (*n* = 5389)	Type 1 diabetes (*n* = 368)	Type 2 diabetes (*n* = 3849)
Understand what metabolic syndrome is (%)	9.6	8.9	11.4	10.4
Have heard about metabolic syndrome (%)	14.1	12.8	14.4	13.8

SHIELD, Study to Help Improve Early evaluation and management of risk factors Leading to Diabetes. Chi-square test, p > 0.05.

### Knowledge, attitudes and behaviours

Because of the low rate of self-reported diagnosis of metabolic syndrome and low awareness or understanding of the syndrome among SHIELD respondents, the high-risk group (3–5 risk factors) was used as a surrogate for metabolic syndrome risk. The high-risk respondents have at least three of the five hallmark risk factors identified by ATP III for metabolic syndrome, including abdominal obesity, general obesity as measured by BMI, dyslipidaemia and hypertension. The high-risk group's responses to questionnaire items that related to health knowledge, attitudes and behaviours were evaluated.

### Knowledge

#### Metabolic syndrome and diabetes

Among the high-risk respondents, lack of knowledge about metabolic syndrome was consistently high across all three attitude/behaviour groups (‘Don’t,’‘Should’ and ‘Doing’). Less than 6% of the high-risk respondents strongly agreed that they had heard about metabolic syndrome and/or understood what it was (Table 4). However, significantly fewer of the ‘Should’ group compared with the ‘Doing’ and ‘Don’t’ groups knew about or understood metabolic syndrome (p < 0.0001). There were significant differences among the groups regarding diabetes knowledge, with the ‘Don’t’ respondents being the least informed (i.e. more likely to agree that diabetes is only a sugar disease and/or that type 2 diabetes is not as serious as type 1) (p < 0.001).

**Table 4:** Health knowledge, attitudes and behaviour among SHIELD high-risk respondents by attitude subgroups (Doing, Should and Don’t)

Topic (%)	Already Doing It (Doing) *n* = 2896† (%)	I Know I Should (Should) *n* = 2149† (%)	Don't Bother Me (Don’t) *n* = 341† (%)
**Knowledge**			
1. I have heard about metabolic syndrome or syndrome X^1^*	5.5	3.5	5.9
2. I understand what metabolic syndrome or syndrome X is^1^*	5.1	2.8	5.1
3. Diabetes is only a sugar disease^1^*	3.8	2.3	12.0
4. Type 2 diabetes is not as serious as type 1 diabetes^1^*	3.4	2.3	8.3
5. The inability to keep weight off is due to a hormone or metabolism problem^1^*	5.2	3.7	11.4
6. Obesity could aggravate or contribute to the onset of chronic diseases^1^*	56.0	39.0	51.9
**Attitude**			
7. I expect my health to get worse^1^*	5.4	5.9	18.0
8. I seem to get sick a little easier than other people^1^*	4.5	4.8	10.1
9. How concerned are you that your health problems will get worse over time?^2^*	27.0	26.3	35.8
10. Would you say your health is excellent, very good, good, fair or poor?^3^*	26.8	38.0	53.5
11. Compared with 12 months ago, how would you rate your health today?^4^*	21.0	25.2	32.8
**Behaviour**			
***Medication taking***			
12. Willing to take medication to prevent chronic disease^1^*	56.4	39.1	47.1
13. Prefer taking medications rather than change my lifestyle^1^*	2.3	3.6	12.8
14. Vary how take prescription medications depending on how I feel^5^*	10.8	16.6	29.9
15. How often refill prescription medications on time?^6^*	92.7	89.8	84.3
16. How often ration medications (skip dose to help prescription last longer)?^7^*	21.6	29.9	32.9
17. Stopped taking medications against doctors’ instructions during the past 12 months^8^*	90.4	87.2	86.9
***Exercise***			
18. Healthcare provider recommended an increase in amount of exercise in past 12 months^8^*	48.9	38.0	43.2
19. Current exercise routine, exercise some or regularly*	78.6	58.6	48.5
***Diet***			
20. Healthcare provider recommended change in diet in the last 12 months^8^*	56.9	48.2	51.0
21. During the last 12 months, have you tried to lose weight?^8^*	25.8	30.4	35.8
22. Have you maintained your desired weight for more than 6 months?^8^*	67.5	75.0	75.1
23. If no, do you believe that it is due to what you eat and how much you exercise?^8^	13.0	11.6	12.5
24. If no, do you believe it is due to an undiagnosed hormone or metabolism problem?*	55.1	55.2	51.3
25. How often do you follow an eating plan prescribed by a physician, nutritionist or dietician?^9^*	17.9	8.2	9.5
26. How often do you try to make healthy choices about what you eat?^9^*	83.1	56.3	48.4
27. How often do you eat breakfast?^9^*	75.0	62.8	59.1
***Alcohol and tobacco behaviour***			
28. Alcohol consumption, ≥ 5 drinks per day in past 4 weeks	7.0	6.7	10.9
29. Smoking history, never smoked*	45.7	45.7	44.3

*p < 0.01 for ANOVA comparison across all three groups. †*n* varies slightly per question.

1 = agree strongly;

2 = very concerned;

3 = fair or poor;

4 = somewhat or much worse;

5 = agree strongly or somewhat;

6 = almost always or always;

7 = a little to all of the time;

8 = no and

9 = most of the time to always.

#### Obesity and weight loss

The high-risk attitude/behaviour groups had significantly different perceptions regarding the impact of a hormone or metabolism problem on weight loss (Table 4). ‘Doing’ and ‘Should’ respondents were less likely to agree strongly that the inability to keep weight off is due to a hormone or metabolism problem (5.2%) and (3.7%) than were ‘Don’t’ (11.4%) respondents (p < 0.0001). More than half of the ‘Doing’ (56.0%) and ‘Don’t’ (51.9%) respondents agreed strongly that obesity could aggravate or contribute to the onset of chronic disease, compared with 39.0% of the ‘Should’ respondents (p < 0.0001).

### Attitudes

#### Health status

The ‘Don’t’ group indicated poorer health status than the ‘Doing’ and ‘Should’ groups (Table 4). More ‘Don’t’ respondents (18.0%) agreed strongly that they expected their health to get worse compared with the ‘Should’ (5.9%) and ‘Doing’ (5.4%) respondents (p < 0.0001). In response to the questionnaire item ‘I seem to get sick a little easier than other people’, 4.5% of the ‘Doing’ respondents agreed strongly compared with 4.8% and 10.1% of the ‘Should’ and ‘Don’t’ groups respectively (p < 0.0001). More than one-quarter of the respondents in all groups were concerned that their health problems would get worse over time, with more ‘Don’t’ respondents being very concerned (35.8%) (p = 0.002).

#### Rating of current health

‘Don’t’ respondents were more likely to rate their health as fair to poor (53.5%), compared with ‘Should’ (38%) or ‘Doing’ (26.8%) respondents (p < 0.0001) (Table 4). When asked about their health in comparison to 12 months ago, about half of all respondents rated their current health as the same. However, fewer ‘Doing’ respondents rated their health as much or somewhat worse than 12 months ago (21.0%) compared with 25.2% of ‘Should’ and 32.8% of ‘Don’t’ respondents (p < 0.0001).

### Behaviours

#### Medication-taking behaviour

A majority of respondents reported a willingness to take medications to prevent chronic disease; however, significantly more ‘Doing’ (56.4%) than ‘Should’ (39.1%) or ‘Don’t’ (47.1%) respondents agreed strongly that they were willing to take medications (p < 0.0001) (Table 4). However, the ‘Doing’ respondents reported the least preference for medication compared with lifestyle changes (2.3%), and ‘Don’t’ respondents reported the highest preference for medication (12.8%) (p < 0.0001). The ‘Doing’ respondents were least likely to vary taking their prescription medications depending on how they felt (‘Doing’, 10.8%; ‘Should’, 16.6%; ‘Don’t’, 29.9%) (p < 0.0001). In addition, more ‘Doing’ respondents reported never rationing their medications (‘Doing’, 78.4%; ‘Should’, 70.1% and ‘Don’t’, 67.1%) (p < 0.0001). Most respondents (> 86%) indicated they had not stopped taking their medications in the last 12 months against their doctor's instruction or approval, and the majority of all respondents (> 84%) said they refilled medication prescriptions on time.

#### Diet, exercise and weight loss

Significant differences were observed when respondents were asked whether a healthcare provider had recommended a change in diet or an increase in the amount of exercise during the prior 12 months (Table 4). More respondents were advised to increase the amount they exercised than were advised to change their diets, with more ‘Should’ respondents receiving this exercise recommendation than ‘Doing’ or ‘Don’t’ respondents (‘Doing’, 51.1%; ‘Should’, 62.0% and ‘Don’t’, 56.8%) (p < 0.0001). Fewer ‘Doing’ respondents (43.1%) than ‘Should’ (51.8%) and ‘Don’t’ (49.0%) respondents reported receiving a recommendation from their healthcare provider to change their diet (p < 0.0001). Significantly more ‘Doing’ (78.6%) respondents than ‘Should’ (58.6%) and ‘Don’t’ (48.5%) respondents reported exercising some or regularly (p < 0.0001).

Among respondents in all groups who indicated an inability to maintain their target weight over the past 6 months, a similar percentage across groups (51–55%) disagreed that a hormone or metabolism problem was responsible for their own inability to lose weight (Table 4). There were no significant differences among the groups regarding their perception of the impact of diet and exercise habits on their inability to maintain their target weight; only 12–13% disagreed that not maintaining desired weight was due to eating and exercise habits.

The majority of respondents (> 60%) in all three groups tried to lose weight in the previous 12 months, but fewer ‘Doing’ respondents than ‘Should’ or ‘Don’t’ respondents reported not trying to lose weight [‘Doing’, 25.8%; ‘Should’, 30.4% and ‘Don’t’, 35.8% (p < 0.0001)] (Table 4). The majority (> 82%) of respondents in all groups were not currently following a prescribed eating plan, although 83.1% of the ‘Doing’ group reported making healthy choices about eating most or all of the time, compared with 56.3% of the ‘Should’ and 48.4% of the ‘Don’t’ respondents (p < 0.0001). Three-quarters (75%) of the ‘Doing’ group reported eating breakfast all or most of the time, compared with approximately 60% of the ‘Should’ and ‘Don’t’ respondents (p < 0.0001).

### Alcohol and tobacco use

There were no significant differences among groups regarding alcohol consumption (Table 4). There were, however, significant differences in smoking habits, with fewer ‘Doing’ respondents (12.9%) reporting that they currently smoke, compared with ‘Should’ (19.2%) and ‘Don’t’ (22.4%) respondents (p < 0.01). In addition, 45.7% of ‘Doing’ and ‘Should’ respondents reported never smoking, compared with 44.3% of ‘Don’t’ respondents.

## Discussion

Study to Help Improve Early evaluation and management of risk factors Leading to Diabetes provides unique insight into US adults’ awareness of metabolic syndrome. In the screening questionnaire, very few respondents (0.6%) self-reported having metabolic syndrome, compared with 25.9% in NHANES. In the baseline questionnaire, an evaluation of the high-risk respondent group who has several components of metabolic syndrome demonstrated that respondents’ knowledge, attitudes and behaviours toward health, exercise and diet varied considerably. When this group of respondents was classified according to their response to the questionnaire item ‘I don't even bother to try and stay healthy’, clear distinctions could be seen between those who reported taking an active approach to improve their health (‘Doing’ respondents) and those who did not (‘Should’ and ‘Don’t’ respondents).

Knowledge was inconsistent within and among the three high-risk attitude/behaviour groups, with respondents generally lacking knowledge about or awareness of metabolic syndrome. Many respondents also did not demonstrate knowledge about diabetes, although most were aware of the impact of obesity on chronic conditions in general.

Respondents’ attitudes toward health were more favourable in those already committed to healthy behaviours (Doing), with poorer health attitudes observed in those who knew they should take better care of themselves (Should) and those who knew it was important, but did not bother (Don’t). The most interesting findings, and perhaps most useful for clinical practice, were the observed associations between these general attitude groups and associated behaviour patterns. Regarding medication-taking behaviour, all respondents were willing to take medications to prevent chronic disease, but the ‘Don’t’ group was the most likely to prefer medication to lifestyle changes. Complicating this as a treatment option, however, was the observation that this group was also the most likely to report changing their medication-taking patterns and rationing their medications. Members of the ‘Don’t’ group would be more difficult to treat because of diet, exercise and medication-taking habits. It is likely that individuals with these characteristics would require substantial support to change their health-related attitudes and behaviours, and they may never embrace such changes. On the other hand, the ‘Doing’ respondents reported adherent medication-taking behaviour and consistently healthy diet and exercise habits. Lastly, respondents in the ‘Should’ group reported a willingness to take medications, but seemed more ambivalent regarding medication-taking vs. lifestyle changes, and reported poorer medication-taking behaviour than the ‘Doing’ group. With appropriate support, the ‘Should’ group may represent the greatest opportunity for successful intervention.

Fifty per cent or more of the ‘Should’ and ‘Don’t’ respondents reported being told by their healthcare provider to exercise more and/or change their diet. Whereas most said they had tried to lose weight, their reported diet and exercise patterns were inconsistent with doing so. The ‘Doing’ respondents also received recommendations for diet and exercise improvements, but reported behaviours were more consistent with those recommendations.

These findings from SHIELD on knowledge, attitudes and behaviours are unique. There is currently little to no information in the literature that has evaluated patients’ health-related behaviour in the context of knowledge and attitudes around metabolic syndrome. However, the connection between knowledge and behaviour has been demonstrated to be tenuous in diabetes (12–14). Therefore, identifying another model that can distinguish patients likely to adopt treatment recommendations from those who are not is important; such a model may help to identify the most appropriate treatment options for patients with different attitudes and knowledge levels. The SHIELD data suggest that respondents who report trying to be healthy are most likely to embrace lifestyle changes and exhibit good medication-taking behaviour, and are less likely to smoke. Respondents who know that treatment and behaviours are important, but do not care, may be the most difficult to treat as they prefer medication over lifestyle changes and are also least likely to take medications appropriately and more likely to smoke. The group in the middle, those who know treatment and behaviours are important but who are not yet taking needed action, may constitute the greatest opportunity for behavioural intervention.

This study provides evidence of the health attitudes and behaviours in a large US population sample of respondents at high risk for metabolic syndrome with a high questionnaire response rate. However, there are limitations to the study that should be considered. Only a small percentage (5–8%) of consumers invited to participate in the TNS panel elect to do so and those who participate are accustomed to completing questionnaires, leading to possible selection bias. Household panels tend to under-represent the very wealthy and very poor segments of the population, and do not include military or institutionalised individuals, which is true for most random sampling and clinically based methodologies. Additionally, the determination of metabolic syndrome and high risk in SHIELD was made based upon self-report rather than clinical or laboratory measures, possibly contributing to the low prevalence of metabolic syndrome reported in SHIELD.

## Conclusions

The lack of knowledge about metabolic syndrome reported in SHIELD suggests that this concept has achieved limited penetration into the public awareness. Given that the actual prevalence of metabolic syndrome is considerably higher than that observed by self-report, increased awareness of the cluster of risk factors defined as metabolic syndrome and education regarding their association with diabetes and CVD risk have the potential to benefit a substantial portion of the US adult population if such awareness can lead to behaviour changes. SHIELD has also provided a unique opportunity to further understand the characteristics of individuals who may be most likely to benefit from health-improving interventions, while also identifying the characteristics of those who have already succeeded, as well as those who may be most difficult to reach.
